# Draft Genome Sequences of *Bordetella hinzii* and *Bordetella trematum*

**DOI:** 10.1128/genomeA.00838-13

**Published:** 2013-10-24

**Authors:** N. R. Shah, M. Moksa, A. Novikov, M. B. Perry, M. Hirst, M. Caroff, R. C. Fernandez

**Affiliations:** Department of Microbiology and Immunology, University of British Columbia, Vancouver, British Columbia, Canadaa; Centre for High-Throughput Biology, University of British Columbia, Vancouver, British Columbia, Canadab; Equipe Endotoxines: Structures and Activities, IGM Université de Paris-Sud, UMR 8621 du CNRS, Orsay, Francec; Immunochemistry Department, National Research Council, Ottawa, Ontario, Canadad

## Abstract

*Bordetella hinzii* colonizes the respiratory tracts of poultry but can also infect immunocompromised humans. *Bordetella trematum*, however, only infects humans, causing ear and wound infections. Here, we present the first draft genome sequences of strains *B. hinzii* ATCC 51730 and *B. trematum* CCUG 13902.

## GENOME ANNOUNCEMENT

The genus *Bordetella* is made up of many host-associated species. *Bordetella pertussis* and *Bordetella parapertussis* are the etiological agents of whooping cough in humans (1), whereas *Bordetella holmesii* is associated with septicemia in humans (2). Other members of this genus are associated with animals, such as *Bordetella bronchiseptica*, found in small mammals, and *Bordetella avium*, a bird pathogen (3). In contrast, *Bordetella petrii* is the only environmentally isolated member of this genus (4). These six species are the previously sequenced members of this genus.

Here, we announce the draft genome sequences of two additional members of the *Bordetella* genus: *Bordetella hinzii* and *Bordetella trematum*. *B. hinzii* colonizes the respiratory tracts of poultry but also has been isolated from immunocompromised humans (5). Alternatively, *B. trematum* has only been isolated from humans and is found to be associated with ear and wound infections (6). *B. hinzii* strain ATCC 57130 was isolated from an immunocompromised person, and *B. trematum* strain CCUG 13902 was isolated from a human leg wound.

The sequencing data for both these strains were obtained from PCR-free random fragment libraries sequenced on the MiSeq (Illumina, Hayward, CA) platform using indexed paired-end 250-nucleotide (nt) v2 chemistry and resulted in ~700-fold coverage for each genome. The nonindexed read length was 250 nt, with 84.4% of the postfilter paired-end reads having Q30 or greater. The sequence reads were subsampled (~2.2 M reads) and assembled into contigs using Velvet (7) with a *k*-mer of 151. A total of 1,850,984/2,212,976 reads were assembled for *B. hinzii* and 1,878,624/2,212,458 reads were assembled for *B. trematum*, resulting in 98 contigs for *B. hinzii* and 83 contigs for *B. trematum*. This is the first report of a draft genome sequence of *B. hinzii* and *B. trematum*.

The genomes were analyzed with the RAST server (8). A total of 4,586 coding sequences were predicted for *B. hinzii*, and 4,145 were predicted for *B. trematum*. For both species, the genes involved with amino acids and their derivatives and those involved with carbohydrates are the major types of predicted genes. Though the overall distributions of the predicted genes among the different subsystem categories are similar between these *Bordetella* species, one notable difference is the greater number of predicted membrane transport genes in *B. hinzii* (308 genes) than in *B. trematum* (184 genes). *B. hinzii* has predicted genes associated with a type II secretion system (T2SS), whereas these genes were not identified in *B. trematum*. *B. avium* (9) and *B. petrii* are the only other *Bordetella* species that have homologs of the same T2SS genes as *B. hinzii* (*gspC* to *gspN*), and these are predicted to produce a functional T2SS by KEGG pathway analysis. This may suggest a closer relationship between the two bird-associated species (*B. avium* and *B. hinzii*) and *B. petrii* (isolated from the environment) in comparison with the other *Bordetella* species.

### Nucleotide sequence accession numbers.

These whole-genome shotgun projects have been deposited in GenBank under accession no. AWNM00000000 (*B. hinzii*) and no. AWNL00000000 (*B. trematum*). The versions described in this paper are the first versions, accession no. AWNM01000000 and AWNL01000000, respectively.
